# Anthropometric characteristics at birth and growth outcome in patients with X-linked hypophosphatemia treated with oral phosphate and active vitamin D

**DOI:** 10.1007/s00467-026-07271-0

**Published:** 2026-04-10

**Authors:** Stephan Przygodda, Laura Celine Brieger, Alina Verena Bohlen, Mirko Rehberg, Martin Konrad, Karl Peter Schlingmann, Olaf Hiort, Dorothee Schmidt, Ulrike John-Kroegel, Elke Wühl, Markus Josef Kemper, Ute Derichs, Ludwig Patzer, Norbert Albers, Desiree Dunstheimer, Sabine Heger, Karina Grohmann-Held, Carmen Schroeder, Norbert Jorch, Elmar Schmid, Hagen Staude, Marcus Weitz, Lena Lecher, Clemens Freiberg, Angela Huebner, Anke Heitmeyer-Pyper, Giuseppina Sparta, Katrina Evers, Anne Juliane Ostendorf, Carl-Joachim Partsch, Michaela Marx, Christof Land, Inka Baus, Frauke Wilkening, Kristina Moeller, Gunter Simic-Schleicher, Susann Empting, Dominik Müller, Oliver Metzing, Verena Wagner, Martin Holder, Mislav Stjepan Žebec, Dirk Schnabel, Dieter Haffner, Miroslav Živičnjak

**Affiliations:** 1 Department of Pediatric Kidney,Liver, Metabolic and Neurological Diseases, Hannover Medical School, Hannover, Germany; 2 Department of Pediatrics, Faculty of Medicine, University Hospital Cologne, University of Cologne, Cologne, Germany; 3 Department of General Pediatrics, Pediatric Nephrology, University Children’s Hospital, Münster, Germany; 4 Division of Pediatric Endocrinology and Diabetes, Department of Pediatrics and Adolescent Medicine, University of Lübeck, Lübeck, Germany; 5 Department of Pediatric Nephrology, University Children’s Hospital, Jena, Germany; 6 Medical Faculty Heidelberg, Center for Pediatric and Adolescent Medicine, Division of Pediatric Nephrology, Heidelberg University, Clinic 1, Heidelberg, Germany; 7 Asklepios Children’s Hospital Hamburg-Heidberg, Hamburg, Germany; 8 University Children’s Hospital, Mainz, Germany; 9 St. Elisabeth and St, Barbara Children’s Hospital, Halle/Saale, Germany; 10 Christliches Kinderhospital Osnabrück, Osnabrück, Germany; 11 Department of Paediatric Endocrinology, Diabetology University Hospital of Augsburg, Augsburg, Germany; 12 Kinderkrankenhaus Auf Der Bult, Hannover, Germany; 13 University Children’s Hospital Greifswald, Greifswald, Germany; 14 University Children’s Hospital, Evangelisches Klinikum Bethel, Bielefeld, Germany; 15 Clinic for Pediatric Nephrology Hirschaid, Hirschaid, Germany; 16 University Children’s Hospital Rostock, Rostock, Germany; 17 Department of General Pediatrics and Hematology/Oncology, University Children’s Hospital Tübingen, Tübingen, Germany; 18 Department of Pediatrics and Adolescent Medicine, University Medical Center Göttingen, Göttingen, Germany; 19 Department of Pediatrics, Faculty of Medicine, University Hospital Carl Gustav Carus, Technische Universität Dresden, Dresden, Germany; 20 Ammersee Ärzte Diessen, Diessen Am Ammersee, Germany; 21 Division of Pediatric Nephrology, University Children’s Hospital Zurich, Zurich, Switzerland; 22 Center for Hormonal and Metabolic Diseases, Reproductive Medicine and Prenatal Medicine, MVZ Endokrinologikum Hamburg, Hamburg, Germany; 23 Pediatric Endocrinology, Children’s Hospital Erlangen, Erlangen, Germany; 24 Child and Adolescent Medicine, Gauting, Germany; 25 University MVZ Kiel, Schleswig-Holstein University Hospital, Kiel, Germany; 26 Helios Children’s Hospital Schwerin, Schwerin, Germany; 27 Department of Pediatrics and Adolescent Medicine, Eltern-Kind-Zentrum Prof. Hess, Klinikum Bremen Mitte, Pediatric Nephrology, Bremen, Germany; 28 Klinikum Bremen-Nord, Bremen, Germany; 29 Department of Paediatric Endocrinology and Diabetology, University Children’s Hospital Magdeburg, Magdeburg, Germany; 30 Department of Pediatrics, Division of Gastroenterology, Nephrology and Metabolic Medicine, Charité-Universitätsmedizin Berlin, Berlin, Germany; 31 Department of Pediatric Endocrinology, University Children’s Hospital, Jena, Germany; 32 Pediatric Practice Rostock - Endocrinology & Diabetology, Rostock, Germany; 33 Klinikum Stuttgart, Children`S Hospital, Pediatric Diabetology, OlgahospitalEndocrinology, Stuttgart, Germany; 34 Institute for Anthropological Research, Gajeva 32, Zagreb, Croatia; 35 Center for Chronically Sick Children, Pediatric Endocrinology, Charité-Universitätsmedizin Berlin, Berlin, Germany

**Keywords:** XLH, Birth anthropometry, Growth, Body disproportion, Hypophosphatemia, Alkaline phosphatase

## Abstract

**Background:**

X-linked hypophosphatemia (XLH) is the most common form of inherited rickets, resulting in short stature despite treatment with oral phosphate and active vitamin D. Detailed data of anthropometric parameters at birth, during infancy, and on the influence of an affected parent on outcome are lacking.

**Methods:**

In this prospective multicenter observational study, conducted from 1998 to 2023 in Germany, Austria, and Switzerland, body length, body weight, and head circumference were investigated in 198 children with XLH from birth until the age of 18 years, all being only on supplementation therapy.

**Results:**

XLH newborns presented with disproportionate body shape characterized by decreased birth length relative to weight and head circumference with a 2.4-fold increased risk to be born small for gestational age (SGA). A positive family history for XLH was associated with lower anthropometric characteristics at birth. Body disproportion increased during 0–2 years old, resulting in significantly increased head circumference (+ 0.82 SD) and reduced body length (−2.00 SD) compared to healthy 2-year-old patients. Supplementation therapy failed to prevent progressive growth failure and reduced final height, regardless of whether treatment was started early due to an affected family member, which was associated with overall poor control of metabolic bone disease indicated by persistent hypophosphatemia and rising alkaline phosphatase *z*-score.

**Conclusions:**

XLH is associated with an increased risk for SGA and progressive disproportional body growth during infancy. Routine medical check-ups may soon use this unique growth pattern to identify children with XLH. Supplemental therapy fails to prevent progressive growth failure.

**Graphical abstract:**

**Figure:**
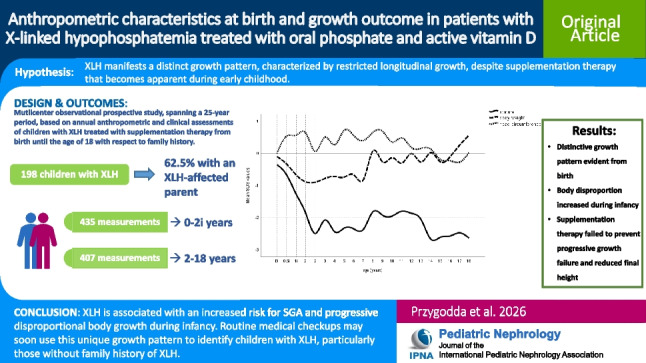
A higher resolution version of the Graphical abstract is available as Supplementary information

**Supplementary Information:**

The online version contains supplementary material available at 10.1007/s00467-026-07271-0.

## Introduction

X-linked hypophosphatemia (XLH) is the most common cause for inherited rickets with an incidence of 3.9 per 100,000 live births and is caused by pathogenetic variants in the *PHEX* (phosphate-regulating endopeptidase homolog X-linked) gene, leading to increased synthesis and secretion of fibroblast growth factor 23 (FGF23) from bone [1]. Elevated levels of FGF23 result in renal phosphate wasting with consecutive hypophosphatemia, rickets, and osteomalacia due to downregulation of the sodium-dependent phosphate transporters NPT2a (encoded by *SLC34A1*) and NPT2c (encoded by *SLC34A3*) and impaired synthesis of 1,25-dihydroxyvitamin D [2]. Patients diagnosed with XLH suffer from disproportionate short stature, mainly due to impaired leg growth or leg bowing [3, 4], as well as bone pain, muscle weakness, and dental abscesses [5, 6]. In early childhood, delayed ability to walk, lower limb deformities, and waddling gait are noticeable, and patients may show premature fusion of the cranial sutures leading to increased prevalence of dolichocephaly [7]. In the absence of alternatives, frequent substitution of high doses of phosphate salts and active vitamin D was the only available therapy for many years, with limited treatment success and being associated with adverse effects including nephrocalcinosis, gastrointestinal discomfort and hyperparathyroidism [7, 8]. Several studies highlighted the positive effects of early initiation of supplementation therapy on growth and rickets activity in XLH patients, especially when started in early infancy [9, 10]. Treatment with burosumab—a fully humanized antibody against FGF23—was shown to be more effective for healing of rickets and resulted in better physical functioning compared to supplementation therapy in children with XLH, while its effects on linear growth was rather moderate [11, 12]. Meyerhoff et al. showed that treatment with recombinant human growth hormone (rhGH) improved growth, but did not normalize adult height nor body proportions in short children with XLH [4].

Newborns with XLH do not exhibit clinical or morphological signs of the underlying pathology [13]. Previous publications on isolated cases or in smaller populations have documented newborns displaying normal birth length and weight. Detailed analyses of anthropometric characteristics at birth including head metrics as well as the influence of an affected parent on growth parameters at birth and outcome predictors are lacking [13–15].

To this end, we analyzed morphological characteristics at birth (body length, body weight, and head circumference), as well as their association with clinical features during the first 18 years of life in 198 children with XLH, treated with phosphate and active vitamin D, who were followed in a prospective multicenter observational study in Germany, Austria, and Switzerland [3, 16].

## Materials and methods

### Study design and patients

From 1998 to 2023, a total of 247 XLH patients were enrolled in a prospective multicenter observational study on the growth of children with XLH at 43 participating centers in Germany, Austria, and Switzerland [3, 16]. The study included children diagnosed with XLH either based on family history and/or genetic confirmation, or based on the presence of clinical and/or radiological signs of rickets, impaired height growth velocity, and serum phosphate levels below the age-related reference range, associated with selective renal phosphate wasting in the absence of vitamin D or calcium deficiency [1]. The study design consisted of two measurement components: (1) annual anthropometric, clinical, and biochemical assessments of XLH patients aged 2–18 years [3] and (2) the anthropometric and birth data taken from the health records for 38.9% to 93.7% of the same XLH patients (depending on the examination time point) within the age range from birth to two years of life.

This analysis was restricted to patients on treatment with phosphate supplements and active vitamin D [16]. Measurements in patients started on burosumab (47.5%) and/or rhGH (12.7%) were excluded from this analysis, to avoid potential artifacts on the growth analysis, as both therapeutic options affect growth [17, 18]. However, the information and measurements prior to the initiation of therapy with latter therapeutics could be incorporated into subsequent calculations. After exclusion of patients on burosumab and rhGH, growth data of 198 patients were analyzed in this paper. Considering repeated measurements of most of the sampled patients (once per year) in the observed period, 407 annual examinations of body length, body weight, head circumference, and biochemical data were carried out in the age range from 2 to 18 years. In addition, assessments including APGAR scores and pH of the umbilical cord at birth (Table 1), as well as 435 measurements (198 patients) of body length, body weight and head circumference from birth through the first two years of life, were taken from the health records. The presence of SGA in XLH newborns was defined as body length and/or body weight below the 10th percentile [19]. The number of assessments taken at birth (B), 6 months (0.5i), 12 months (1i), and 24 months (2i) amounted to 177, 88, 93, and 77, respectively. These time points correspond to the recommended or legally required standardized childhood development examinations U1, U5, U6, and U7 [20], carried out in the participating countries. Patients and/or their parents provided written informed consent to participate in this study. The study was approved by the Ethics Committee of the Hannover Medical School (No. 7259) and by the Ethics Committees of the individual participating centers; it was conducted in accordance with the ethical standards of the Declaration of Helsinki of 1964 and its subsequent amendments.

**Table 1:** Relevant clinical, physical and family characteristics of 198 patients with X-linked hypophosphatemia (XLH)

Relevant clinical and family characteristics^a^	***Incidence/MV***	***No. of cases/measurements***	***p-value***
Female sex	60.6%	120 of 198	0.001
Affected parent	62.5%	121 of 195^b^	< 0.05
- Mother affected	75.2%	91 of 121	< 0.001
Mother height, cm	160 (150 to 168)	195 of 198	
Father height, cm	176 (172 to 181)	195 of 198	
Age at diagnosis, years	1.79 (0.29 to 3.02)	185 of 198	
Age at start therapy, years	2.14 (0.90 to 3.76)	167 of 198	
Menarcheal age, years	12.61 (11.84 to 14.05)	27 of 120	
*PHEX* mutation, %	87.0	100 of 115^b^	
SGA, %	19.6	32 of 163^b^	
Birth data^a^	***Median (IQR)***	***No. of cases/measurements***^c^	
Gestational age, weeks	39.4 (38.3 to 40.0)	163 of 177	
Birth length, SDS	−0.23 (−0.69 to 0.34)	175 of 177	
Birth weight, SDS	−0.12 (−0.86 to 0.67)	177 of 177	
Birth head circumference, SDS	0.24 (−0.65 to 0.72)	157 of 177	
APGAR, 5‘/10‘	10 (9 to 10)/10 (10 to 10)	155/155 of 177	
pH, umbilical cord	7.28 (7.23 to 7.38)	123 of 177	

MV, measured value; ± SD, standard deviation, IQR, interquartile range; SDS, standard deviation score; *PHEX*, phosphate-regulating endopeptidase homolog X-linked gene; SGA, small for gestational age; APGAR, appearance, pulse, grimace, activity, respiratory/respiration, score after 5 and after 10 min postpartum

*p*-value relates to comparison with normative data

^a^Descriptive data (nonrepeated measurements) are given as Incidences %, mean ± SD or median and IQR (percentiles: 25th and 75th), depending on distribution of normality of data

^b^Nonrepeated measurements of all 198 patients. In case of a number less 198, no further measurements were available

^c^At the time of birth, a maximum of 177 measurements were available

### Methods

In the age group from birth to 2.0 years, age- and sex-specific *z*-scores for anthropometric measures were calculated using German reference data for healthy children aged from birth to 2.0 years [21]. The following age group intervals were used for this purpose: birth = 0.00 to 0.003 years, 0.5i = 0.458 to 0.541 years, 1.0i = 0.958 to 1.042 years, and 2.0i = 1.875 to 2.124 years. Patients were classified into two subgroups based on their family history: patients born to healthy parents and those whose parents were affected by XLH (mother or father).

Anthropometric assessments (407 measurements of stature, body weight, and head circumference) from the 2nd year of life onward were performed annually by the same investigator (MŽ), as recommended by the International Biological Program [22] with standardized equipment. *Z*-scores were calculated using reference data of 5260 healthy children [23, 24]. Standard laboratory procedures were used for biochemical indicators gathered at the annual measurement including serum hemoglobin and phosphate, calcium, alkaline phosphatase (ALP), creatinine, parathyroid hormone (PTH), and 25OH vitamin D. Hypophosphatemia was classified and *z*-scores were calculated for serum phosphate, calcium, ALP, and PTH values using age- and sex-specific reference values utilized as already employed in previous studies [16, 25, 26]. Estimated glomerular filtration rate (eGFR) was calculated by use of the revised Schwartz equation [27]. The age at diagnosis and the age at menarche were taken from the patient's personal health records or medical history. Genetic analyses were conducted for the presence of *PHEX* mutations in 115 of the 198 patients. As part of the long-term study, genetic testing was not always performed for diagnostic confirmation, particularly in participants who were enrolled in the study at a very early stage; probably due to the high costs associated with the procedure at this time, as well as regional limitations in availability. Annual measurements included screenings for the presence of nephrocalcinosis as part of kidney ultrasound examinations.

### Statistical analysis

Patients’ measurements within the first two years of life (health care records) were clustered into four age groups (birth, 0.5i, 1.0i, and 2.0i), while measurements from 2 years of age onwards were clustered into three age groups (2–6 years, 7–12 years, and 13–18 years) corresponding to A = early childhood (*n* = 110), B = late childhood, prepubertal and early pubertal age (*n* = 184), and C = adolescence (*n* = 113), respectively [28–30].

**Fig. 1:**
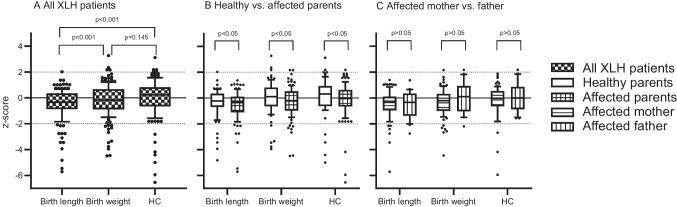
Birth anthropometry in newborns with XLH. Mean SDS values of birth anthropometric measurements which were taken from the corresponding health records in neonates with XLH. The individual boxes with the corresponding whiskers represent the SDS values of birth length, birth weight and head circumference between the 10th and 90th percentiles. Each point represents an additional measurement outside this range (outlier). The single transverse line within each box represents the median of the measurements. HC: Head circumference. (**A**) Birth length, birth weight, and head circumference (No.: 175/177/157) in all XLH patients. Median values: birth length: −0.23; birth weight: −0.17; and head circumference: 0.24. (**B**) Birth length, birth weight and head circumference with respect to family history of XLH and after clustering in groups of children with healthy parents and affected parents (No.: 77/121). Median values healthy/affected: birth length: 0.00/−0.34; birth weight: 0.19/−0.18; and head circumference: 0.31/0.00. (**C**) Birth length, birth weight, and head circumference with respect to whether the mother or the father (No.: 85/20) is the carrier of the disease. Median values: birth length: (−0.34/−0.34); birth weight: (−0.21/0.10); and head circumference: (−0.03/0.00)

**Fig. 2:**
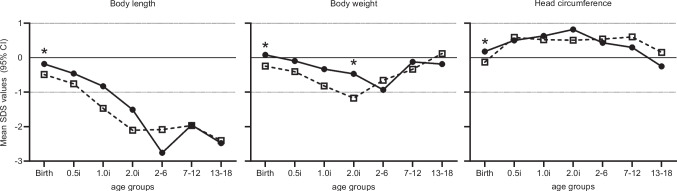
Dynamics of growth from birth to adolescence in 198 patients regarding their family history. Mean SDS values of body length, body weight, and HC respect to age groups and family history of 198 patients with XLH. Age in years. Circle: patients without XLH-affected parents. Square: patients with XLH-affected parents. The asterisk marks the age groups with statistically significant differences. Measurements of birth, 0.5i, 1.0i, and 2.0i were taken from health care booklets. Measurements of the older age cluster were taken from annual anthropometric measurements

**Fig. 3:**
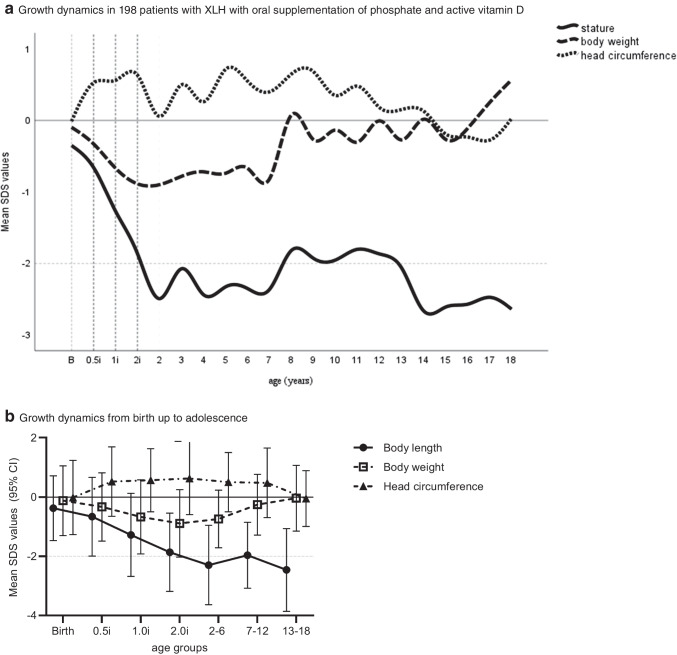
(**a**) Growth dynamics in 198 patients with XLH with oral supplementation of phosphate and active vitamin D. Body length or stature, body weight, and head circumference in children with XLH. B: birth; 0.5i, 1i, and 2i: age of infant in years. Information from birth up to age group 2i gathered from personal health records. From age group 2 onwards, SDS values were calculated out of self-performed anthropometric measurements. Growth dynamics from birth up to adolescence. (**b**) Dynamics of growth in 198 patients with XLH with oral supplementation with phosphate und active vitamin D presented by mean SDS values of body length, body weight and head circumference. Measurements of birth, 0.5i, 1.0i and 2.0i were taken from the personal health-records, while the ones of the older age groups were taken from annual anthropometric measurements

Descriptive statistics related to patients’ disease relevant clinical, physical, and family characteristics (e.g. female sex, parental height, or age of diagnosis) are given through percentages (incidence), mean and standard deviation (SD), or median and interquartile range (IQR), as appropriate (Tables 1 and 2). These statistics are also given as minimal and maximal values, estimated marginal means and confidence intervals (CI) (Tables 2 and 3). Lastly, the description of anthropometric measures at birth and their later age-related changes is given through figures (Figs. 1, 2, 3a, and 3b).

**Table 2:** Birth-relevant, clinical, and anthropologically relevant characteristics of patients with X-linked hypophosphatemia according to their family history

*Nonrepeated measurements*^*a*^	***Without affected parents***			***Affected parents***			*p-value*
	*Mean (*± *SD)/Median (IQR)*	*Min.–Max*	*N*	*Mean (*± *SD)/Median (IQR)*	*Min.–Max*	*N*	
Birth data, SDS values							
- Birth length	0.00 (−0.55 to 0.42)	−4.83 to 2.41	67	−0.34 (−1.03 to 0.22)	−5.71 to 1.39	108	0.019
- Birth weight	0.19 (−0.54 to 0.91)	−3.95 to 3.27	69	−0.18 (−0.95 to 0.37)	−4.46 to 2.35	108	0.031
- Head circumference	0.31 (−0.31 to 0.94)	−5.00 to 3.13	60	0.00 (−0.65 to 0.53)	−5.94 to 2.19	97	0.044
APGAR							
- After 5 min	10 (10 to 10)	7 to 10	59	10 (9 to 10)	5 to 10	95	0.090
- After 10 min	10 (10 to 10)	8 to 10	59	10 (10 to 10)	7 to 10	95	0.510
pH	7.29 (7.23 to 7.36)	7.07 to 7.46	44	7.28 (7.23 to 7.32)	7.01 to 7.43	78	0.432
Gestational age, weeks	40.0 (39.0 to 40.0)	28.6 to 41.0	62	39 (38.0 to 40.0)	28.0 to 42	97	0.124
SGA, %	17.2		11/64	21.2		21/99	0.531
Age at diagnosis, years	2.9 (2.03 to 3.96)	0.0 to 15.2	69	0.61 (0.05 to 2.15)	0.0 to 11.0	116	< 0.001
Age at start therapy, years	2.9 (2.14 to 4.12)	0.0 to 14.5	63	1.17 (0.44 to 2.91)	0.0 to 13.3	93	< 0.001
Parental height							
- Mother, cm	167.0 (162.0 to 170.0)	147.0 to 181.5	74	153.5 (149.0 to 160.0)	134.1 to 173.0	121	< 0.001
- Father, cm	180.0 (173.0 to 183.3)	159.0 to 203.0	74	176.0 (168.5 to 180.0)	140.0 to 190.0	121	< 0.001
PHEX, %	77.1		27/35	91.3		73/80	0.058
Age at menarche, years	12.9 (± 1.5)	10.9 to 16.0	12	13.0 (± 1.3)	11.2 to 15.7	15	0.830

SDS, standard deviation score, ± SD, standard deviation; IQR, interquartile range

^a^Descriptive data (nonrepeated measurements) given as mean ± SD or median and IQR (percentiles: 25th and 75th), depending on normality of data distribution

**Table 3:** Biochemical characteristics of 198 XLH patients categorized by age cohorts corresponding to early (Group A) and late (Group B) childhood and adolescence (Group C) and compared via linear mixed effects model analysis or indicated as incidences

	***2 to 18 years***	***Group A (2 to 6 years)***	***Group B (7 to 12 years)***	***Group C (13 to 18 years)***
*Repeated measurements*^*a*^	*E. marginal mean* *(95% CI)*	*E. marginal mean* *(95% CI)*	*E. marginal mean* *(95% CI)*	*E. marginal mean* *(95% CI)*
Age, years	10.17 (10.01 to 10.33)	4.92 (4.62 to 5.22)^B**C**^	9.98 (9.74 to 10.21)^A**C**^	15.61 (15.31 to 15.91)^A**B**^
Hemoglobin, g/dl	13.22 (13.01 to 13.42)	12.51 (12.18 to 12.84)^B**C**^	13.31 (13.07 to 13.54)^A**C**^	13.83 (13.56 to 14.10)^A**B**^
Phosphate, *z*-score	−3.12 (−3.40 to −2.85)	−2.79 (−3.17 to −2.40)^B*C*^	−3.19 (−3.52 to −2.87)^A*^	−3.40 (−3.80 to −3.00)^A*^
Calcium,* z*-score	−0.31 (−0.47 to −0.16)	−0.30 (−0.53 to −0.06)^C**^	−0.33 (−0.52 to −0.13)^C**^	−0.31 (−0.56 to −0.06)^A**B**^
ALP, *z*-score	2.79 (2.37 to 3.01)	2.65 (2.24 to 3.07)	2.34 (1.98 to 2.71)^C**^	3.08 (2.64 to 3.51)^B**^
PTH, *z*-score	1.82 (1.52 to 2.11)	1.50 (1.07 to 1.93)^C*^	1.59 (1.23 to 1.95)^C*^	2.36 (1.91 to 2.82)^A*B*^
25OH vitamin D, ng/ml	38.32 (28.74 to 47.90)	41.79 (27.91 to 55.68)	36.62 (24.60 to 48.64)	36.54 (18.23 to 54.85)
eGFR, ml/min per 1.73 m^2^	118.91 (113.75 to 124.07)	127.18 (120.34 to 134.03)^B**C**^	120.01 (114.07 to 125.95)^A** C**^	109.55 (102.36 to 116.73)^A**B**^
Phosphorus (mg/kg/day)	52.74 (47.02 to 58.47)	56.37. (49.18 to 63.57)^C*^	54.05 (47.63 to 60.48)^C*^	47.81 (40.64 to 54.98)^A*B*^
Calcitriol dosage (ng/kg/day)	20.04 (17.74 to 22.33)	22.63 (19.70 to 25.57)^C**^	20.26 (17.68 to 22.85)^C*^	17.21 (14.36 to 20.07)^A**B*^
	*-*	*Incidence (N)*	*Incidence (N)*	*Incidence (N)*
Nephrocalcinosis, in %	-	36.4 (16 of 44)^C*^	39.1 (34 of 87)^C*^	57.2 (36 of 63)^A*B*^

ALP, alkaline phosphatase; PTH, parathyroid hormone

^a^Repeated measurements and estimated marginal means with 95% confidence intervals within the same individuals, depending on annual measurements; *p < 0.05 and **p < 0.01, i.e., the measured hemoglobin serum value increases significantly from age group A to B and B to C with a *p*-value of *p* < 0.01

A set of inferential statistical procedures was conducted on measured data. The Kolmogorov–Smirnov test with and without Lilliefors correction and the Shapiro–Wilk test were used to evaluate normality of distribution. Depending on the distributions’ normality, either Mann–Whitney test or *t*-test was used to test differences between groups of mostly anthropometric measurements between patients with and without XLH affected parents, or between patients with affected mother and affected father, on the whole sample of patients (Table 2), or at birth (Fig. 1B and C), or within each age cohort (Fig. 2; from birth to 2.0i). The Chi-square test was used to investigate differences in the frequency of the characteristics measured on a nominal scale of the patients (e.g., affected parent, Table 1).

Pairwise comparisons from linear mixed-effects models (LMM) were used to test differences among patients’ clinical and anthropometrical characteristics measured at three separate age clusters (groups A, B, and C at Table 3) or at birth (Fig. 1 A).

Body length, body weight, and head circumference growth were analyzed separately in patients with and without a family history for XLH, across seven age cohorts: birth, 0.5i, 1.0i, 2.0i, 2–6 years, 7–12 years, and 13–18 years (Fig. 2). Additionally, we proceeded to analyze the pattern of growth at the entire patient sample irrespective of their family history of the disease, as growth data did not differ according to family history after the infancy period (Fig. 3a and b).

LMM were also used to analyze which clinical characteristics are associated with the observed anthropometric measures within the different age groups (Table 4). Anthropometrical data were adjusted for covariates, i.e., age, parental height, age at diagnosis, hemoglobin, phosphate, calcium, ALP, and PTH and eGFR. Consequently, we examined various covariate structure models and selected the optimal model based on the information criteria specific to each analysis and parameter group.

**Table 4:** Linear mixed-effect models of clinical determinants of body height, weight, and head circumference in XLH patients categorized by age cohorts

	***Group A*** ***(2 to 6 years)***	***Group B*** ***(7 to 12 years)***	***Group C*** ***(13 to 18 years)***
*Body height*			
Intercept	−5.54 (−15.78 to 4.71)	−5.30 (−9.57 to −1.03)*	−11.59 (−20.60 to −2.58)*
Age	−0.18 (−0.43 to −0.07)	−0.01 (−0.10 to 0.08)	0.31 (0.06 to 0.56)*
Parental height	0.70 (−1.38 to 2.79)	1.96 (1.18 to 2.74)**	2.06 (0.09 to 4.04)*
Age at diagnosis	−0.38 (−0.72 to −0.04)*	−0.27 (−0.39 to −0.14)**	−0.11 (−0.26 to 0.04)
Hemoglobin	0.49 (−0.09 to 1.06)	−0.06 (−0.30 to 0.19)	−0.21 (−0.56 to 0.14)
Phosphate, *z*-score	0.43 (−0.01 to 0.87)^0.053^	0.16 (0.07 to 0.26)**	−0.13 (−0.34 to 0.07)
Calcium, *z*-score	−0.28 (−0.66 to 0.10)	0.02 (−0.14 to 0.19)	0.36 (0.09 to 0.63)*
ALP, *z*-score	−0.07 (−0.26 to 0.12)	−0.12 (−0.27 to 0.03)	−0.10 (−0.29 to 0.09)
PTH, *z*-score	−0.36 (−0.57 to −0.15)**	0.002 (−0.10 to 0.10)	0.26 (0.04 to 0.48)*
eGFR (× 10)	−0.08 (−0.20 to 0.04)	−0.04 (−0.09 to 0.01)	−0.03 (−0.14 to 0.20)
*Body weight*			
Intercept	12.17 (3.50 to 20.84)*	−1.04 (−5.17 to 3.09)	−4.46 (−10.96 to 2.04)
Age	−0.27 (−0.45 to −0.08)**	−0.02 (−0.11 to 0.07)	0.16 (−0.06 to 0.37)
Parental height	−1.39 (−3.05 to 0.27)	0.25(−0.51 to 1.0)	0.70 (−0.67 to 2.06)
Age at diagnosis	0.07 (−0.19 to 0.33)	−0.04 (−0.16 to 0.09)	−0.19 (−0.31 to −0.06)**
Hemoglobin	−0.40 (−0.93 to 0.13)	−0.02 (−0.26 to 0.21)	0.00 (−0.26 to 0.26)
Phosphate, *z*-score	0.59 (0.20 to 0.98)**	−0.10 (−0.20 to −0.01)*	0.01 (−0.15 to 0.16)
Calcium, *z*-score	−0.08 (−0.35 to 0.20)	0.03 (−0.14 to 0.20)	0.04 (−0.16 to 0.24)
ALP, *z*-score	0.03 (−0.11 to 0.17)	−0.11 (−0.26 to 0.04)	−0.02 (−0.17 to 0.14)
PTH, *z*-score	−0.25 (−0.40 to −0.09)**	0.03 (−0.07 to 0.13)	0.09 (−0.07 to 0.25)
eGFR (× 10)	−0.03 (−0.14 to 0.07)	0.02 (−0.03 to 0.07)	−0.01 (−0.14 to 0.13)
*Head circumference*			
Intercept	−1.43 (−9.01 to 6.14)	5.20 (1.10 to 9.28)*	−2.24 (−3.10 to 7.59)
Age	−0.18 (−0.39 to 0.03)	0.06 (−0.03 to 0.14)	−0.001 (−0.15 to 0.15)
Parental height	−0.68 (−2.26 to 0.91)	−1.73 (−2.48 to −0.99)**	−0.63 (−1.80 to 0.54)
Age at diagnosis	0.10 (−0.16 to 0.37)	0.08 (−0.04 to 0.20)	−0.13 (−0.22 to −0.04)**
Hemoglobin	0.29 (−0.12 to 0.69)	−0.03 (−0.26 to 0.20)	−0.004 (−0.21 to 0.23)
Phosphate, *z*-score	−0.15 (−0.47 to 0.17)	0.13 (0.05 to 0.22)**	−0.03 (−0.15 to 0.09)
Calcium, *z*-score	−0.19 (−0.53 to 0.14)	0.01(−0.14 to 0.17)	0.04 (−0.13 to 0.20)
ALP *z*-score	−0.06 (−0.21 to 0.10)	0.22 (0.08 to 0.36)**	−0.13 (−0.239 to −0.02)*
PTH (× 10), *z*-score	−1.69 (−3.38 to 0.00)^0.05^	−0.42 (−1.32 to 0.48)	1.24 (−0.05 to 2.53)
eGFR (× 100)	0.70 (−0.20 to 1.50)	−0.03 (0.08 to 0.02)	−1.30 (−0.70 to 0.07)

Data are presented as β-values (95% confidence intervals). Patients were examined several times within age groups. For estimations of effects of calcium, phosphate, alkaline phosphatase and parathormone *z*-scores were used

Algebraic sign in *β*-values expresses positive (+) or negative (–) association, e.g., “−0.38” for Age at diagnosis in the age cluster 2–6 years represents earlier age of diagnosis to be associated with higher body length

*p < 0.05, **p < 0.01

Statistical significance was defined as a *p*-value below 0.05. SPSS for Windows, version 28.0 (IBM Corporation, NY, USA) was used for statistical analyses and to generate Fig. 3a. GraphPad Prism 9.3.1 (GraphPad Software Inc., San Diego, CA, USA) was used to create Figs. 1, 2, and 3b.

## Results

This analysis included 198 pediatric patients with XLH, mostly female (60.6%), with mostly positive family history (62.5%) and transmission through the mother (75.2%, each *p* < 0.05; Table 1). Pathogenic variants of the *PHEX* gene were detected in 87.0% of tested patients and documented at a higher prevalence in children with affected parents (91.3%) compared to children with non-affected parents (77.1%, *p* = 0.058; Table 2). The median age at diagnosis and start of treatment with phosphate supplements and active vitamin D amounted to 1.79 years (IQR 0.29–3.02) and 2.14 years (IQR 0.9–3.76), respectively; both were significantly lower in patients with a family history of XLH compared to patients without family history (0.61 years and 1.17 years versus 2.9 years and 2.9 years, each *p* < 0.05; Table 2). Parental height was reduced to 160 cm in mother and to 176 cm in father (Table 1) and was significantly lower in patients with one affected parent (Table 2). The daily dosages of the supplementation therapy during the observation period, based on elemental phosphorus and calcitriol, were significantly lower in adolescence compared to early childhood (56.37 mg/kg vs. 47.81 mg/kg; *p* < 0.05; Table 3). During the same time period, an increase in ultrasound-assisted diagnosis or a deterioration of an already known nephrocalcinosis was observed (36.4% vs. 57.2%; *p* < 0.05; Table 3). Estimated marginal mean of phosphate *z*-score (−3.1) was reduced and significantly decreased across the age groups (each *p* < 0.05; Table 3). ALP (2.79) and PTH (1.82) *z*-scores were increased, both continued to increase from early childhood to adolescence (both *p* < 0.05). Calcium *z*-score (−0.31) was in the entire observation period in the lower normal range; however, our analyses indicated a statistically significant decrease with higher age (Table 3). A significant increase in hemoglobin and a significant reduction in kidney function were observed at the transition between the age groups, albeit within the normal range (Table 3).

### Birth data

Overall, the median gestational age, length, weight, and head circumference in newborns with XLH were comparable to those of healthy newborns (Table 1 and Fig. 1A); nevertheless, 19.6% of them were classified as SGA, irrespective of a positive family history for XLH (17.2% with healthy versus 21.2% with affected parents, *p* = 0.53; Table 2), which was 2.4-fold higher than in the general German population (8.1%; *p* < 0.001) [19]. The further analysis indicated statistically significant differences between the standardized values (*z*-scores) of body length and head circumference (−0.23 vs. 0.24; *p* < 0.05; Table 1 and Fig. 1A). At birth, patients with an affected parent presented significantly smaller, lighter and with a reduced head circumference (each *p* < 0.05; Table 2 and Fig. 1B), when compared to patients without family history of XLH. However, in the group of patients with a family history of XLH, there was no significant morphological difference depending on the carrier of the disease (mother or father; Fig. 1C). For this purpose, we were able to examine the morphological characteristics of newborns whose mother or father was a carrier of the disease (affected mother/affected father; *N* = 85/20; Fig. 1C). Furthermore, median gestational age, APGAR score after 5 and 10 min, as well as umbilical cord pH, did not differ significantly between groups with and without affected parents (each *p* > 0.05; Table 2), nor were there any differences within the group of patients with affected parents.

### Growth at the first two years (0–2 years)

In the total study population, median body length and weight *z*-scores decreased significantly at the first 2 years (birth to 2i) from −0.23 to −2.00 and from −0.12 to −0.98, respectively, while head circumference *z*-score increased significantly from 0.24 to 0.82 at 2 years (each *p* < 0.01, Fig. 3a and b). These differences in growth of body length and head circumference resulted in a progressive growth disparity pattern during early life age. Body length *z*-scores converged in patients with and without an affected parent during the first two years of life since the significant difference at birth disappeared during infancy. The former was due to a significantly steeper slope of the body length *z*-score in patients without an affected parent referring to the entire observation period (birth to 2i; *p* < 0.05). In contrast, median weight *z*-score was still greater in patients without an affected parent at the age of 2 years (−0.47 vs. −1.17; *p* < 0.05).

### Growth in childhood and adolescence (2–18 years)

Overall, the body length deficit that already existed at the age of 2 years did not improve under treatment with supplementation therapy during the growth period (Fig. 3a and b). The entire study sample presented a significant increase in height *z*-score between the age group 2 and 6 compared to 7 and 12 years (−2.37 vs. −2.10; *p* < 0.001), and a decline to 13 and 18 years (−2.44; *p* < 0.001; vs. ages 7–12 years; Fig. 3b). The mean *z*-scores of body weight increased differently between the ages of 2 and 6 years compared to 7 and 12 years and 13 and 18 years (−1.0 vs. −0.55 and −0.41, respectively; each *p* < 0.01). Head circumference *z*-score of patients with XLH was constantly higher than in healthy children throughout the whole observed developmental period (*p* < 0.01) and normalised between ages 13 and 18 years (Figs. 3a, b). Notably, there was no significant difference in anthropometric characteristics in patients with and without family predisposition between 2 and 18 years (each *p* > 0.05; Fig. 2).

It is worth noting that

i. Standardized body height values were lowest in the 13–18 age group, although the difference between ages 2 and 6 years compared to 13 and 18 years was not statistically significant (−2.37 vs. −2.44; *p* = 0.41).
ii. The only anthropometric parameter that significantly differed between birth and the end of growth was the z-score for body length/height (−0.23 vs. −2.44; *p* < 0.001; Fig. 3a and b).
iii. In parallel with this, the *z*-scores for body weight and head circumference at birth were not significantly different to those in the 13–18 age group (−0.12 vs. −0.41 and −0.24 vs. −0.29, respectively).

### Clinical predictors of height, weight, and head circumference *(*Table 4*)*

Higher phosphate *z*-score and younger age of disease diagnosis were associated with better growth in XLH patients aged 2 to 12 years; at the age of 2–6 years, the association between phosphate *z*-score and body height did not reach a statistical significance level (*p* = 0.05; Table 4). Better statural growth in late prepubertal and adolescent XLH children (aged 7 to 18 years) was positively associated with parental height. Conversely, lower PTH *z*-scores from ages 2 to 6 years but higher scores from ages 13 to 18 years were linked to better growth in children with XLH. Significant (positive) associations between stature and calcium *z*-scores were only seen within the oldest age group.

On the other hand, body weight was more closely linked to clinical parameters at the age of 2–6 years than at any other point in growing age. In this age cluster, younger patients were expected to have higher weight. Also, patients with higher serum phosphate and low PTH *z*-scores tended to have significantly higher body weights.

A statistically significant association has been found between lower phosphate *z*-scores at age 7–12 and earlier ages of XLH diagnosis in children aged 13–18, with higher body weight *z*-scores.

Within the 2–6 age group, the only borderline association was found to be between the z-value of PTH and head circumference (negative; *p* = 0.05). Children aged 7–12 with lower parental height and higher phosphate and ALP *z*-scores had larger head circumferences. Larger head circumference in adolescence (aged 13–18 years) was associated with lower ALP values and an earlier age of diagnosis.

## Discussion

This multicenter prospective observational study of 198 XLH patients conducted from 1998 to 2023 presents anthropometrical parameters at birth, infantile and post-infantile growth and the effects of a family history and thus earlier treatment with phosphate salts and active vitamin D on outcome. Our data show that XLH results in a 2.4-fold increased risk to be born SGA, and body disproportion already at birth, with decreased birth length relative to weight and head circumference, which progressively increased during infancy. Newborns with XLH and one affected parent have smaller birth metrics than those without an affected parent. However, this difference is no longer observable by the end of early childhood. Finally, treatment with oral phosphate and active vitamin D fails to prevent progressive growth failure and reduced final height.

The anthropometric parameters at birth appeared normal in patients with XLH, which is in line with previous studies reporting normal body length and weight in newborns with XLH [13, 14]. However, in the present study, newborns with XLH showed a statistically significant reduction in body length in relation to head circumference, indicating body disproportion at birth, which has a multifactorial intrauterine origin. Therefore, our findings showed a 2.4 times higher frequency of SGA in our patient cohort compared to the German general population (19.6% vs. 8.1%). FGF23-mediated hypophosphatemia is a major cause of growth impairment in children with XLH in postnatal life; XLH may affect intrauterine growth as well [2, 31, 32]. As treatment with oral phosphate and active vitamin D further stimulates FGF23 levels in XLH patients, this may at least partly explain why treatment with phosphate supplements and active vitamin D does not prevent progressive growth failure during infancy and later life [33]. Impaired fetal growth may be an effect of the mutated *PHEX* gene, which is expressed in proliferating and hypertrophic chondrocytes [2, 31].

The discrepancy between body length and head circumference progressively increased during the first two years of life, resulting in significantly increased head circumference and reduced body length compared to healthy children. Already six months after birth, there was a distinctive change in the difference between these *z*-scores, reaching a difference of 1.19 *z*-score. This observed inverse pattern between linear and head growth during infancy appears to be a specific feature of XLH in children and could be used in the future to identify children with XLH earlier in routine medical check-ups. This seems to be of particular importance in case of a negative family history, as the average age of diagnosis in this group amounted to 2.9 years in the present study, which is in line with previous reports [34]. Indeed, an Italian multicenter prospective expert survey of 175 XLH patients showed that only 11% of children were diagnosed at age 0–1 years, 50% at age 1–5 years, and even 14.7% of patients at 12 years or older [35].

In contrast to impaired linear growth, head circumference progressively increased during infancy in the present study which may be the consequence of altered skull morphology, i.e., rickets-associated frontal bossing and or subclinical scaphocephaly noticed in patients with XLH [36, 37]. Dolichocephaly is caused by premature fusion of the cranial sutures (i.e., craniosynostosis) probably due to crossbinding of elevated FGF23 to FGFR2 and FGFR3 at the cranial sutures in infants with XLH [2, 38, 39].

The age-related changes and growth of the head are characterized by specificity and complexity, stemming from a combination of multiple clinical and physiological factors. Consequently, it is anticipated that, in early childhood, apart from PTH (with borderline significance), no other associations were identified with the assessed parameters. A lower parental height has been found to be associated with a larger head circumference in patients aged 6 to 12 years. The extent to which higher serum levels of phosphate and ALP in this age group and lower levels in adolescence are associated with larger head circumference remains unclear.

Interestingly, newborns with XLH with an affected parent were significantly shorter, lighter and had a reduced head circumference than those without an affected parent, regardless of which parent was affected. This could be at least partly due to the significantly shorter stature of the affected parents, since the parents’ height is associated with their children’s height at birth in the healthy population [40–42]. However, birth length is also determined by additional factors, such as the width of the mother’s pelvis [43]. In line with this, we could previously demonstrate that female adolescents with XLH show relatively well-preserved pelvic width (−0.32 *z*-score) [16]. Therefore, the newborn is not expected to be much shorter. This is related to our findings that there is no significant difference in the observed morphology of newborns of affected mothers compared to affected fathers (Fig. 1 C); although mothers (XLH carriers) were statistically significantly shorter than healthy mothers who had an XLH-positive child with XLH-positive father (152 cm vs. 163 cm; *p* < 0.01).

However, the effectiveness of supplemental therapy in ameliorating progressive growth failure remained unsatisfactory in the vast majority of patients, regardless of whether treatment was started early due to an affected family member. Notably, in late childhood, there was a slight catch-up growth under this therapy, but this was more than negated by a significant drop in standardized height after the age of 13 years—the time of expected pubertal growth spurt [3, 7, 44]. This may be partly explained by the overall poor control of metabolic bone disease demonstrated by persistent hypophosphatemia and markedly increased ALP *z*-scores indicating increased osteoblast activity and ongoing rickets and osteomalacia in the face of impaired growth.

Cole’s concept of the positive secular trend of healthy children can be used to explain the dynamics of XLH-specific growth restriction: “Most of the increase in height in adulthood occurred by the age of 1.5 years, suggesting that the secular trend in height represents increased growth of long bones during infancy” [45]. Contrary to this positive trend observed in healthy children, the most pronounced growth deficit was observed in the first two years in our study population, which may explain the markedly impaired stature observed after growth has completed. The leg length of XLH patients is most affected at the age of two [3]. This seems to be the primary factor contributing to stunting throughout the growth period until adolescence.

During pubertal age, ALP *z*-scores increased from 2.34 to 3.08 *z*-score, accompanied by markedly impaired linear growth, which may be partly related to reduced adherence to therapy during this challenging time period. Further, markedly elevated ALP *z*-scores despite continuous treatment with oral phosphate and active vitamin D were previously reported in pediatric XLH patients [46]. Astonishingly, we could not support its negative impact on linear growth, as there were no significant associations of ALP *z*-scores from ages 2 to 18 years regarding statural growth and body weight development. In contrast, earlier diagnosis and higher phosphate levels had a distinctive impact on statural growth between the ages of 2 and 12 years. Consistent with the better slope in children who were diagnosed earlier, due to either being more severely affected by XLH and presenting noticeable growth impairments or having affected parents, these patients receive earlier treatment, and the age at diagnosis thus serves as a clinical predictor for better growth in children aged 2 to 12 years.

Regarding the parental height, this only affects the stature from later childhood onwards, whereas no association could be determined in early childhood. It can be assumed that other additional parameters not examined by us may show associations, or that body length is already fixed in infancy and a growth spurt corresponding to parental height occurs during puberty.

The reversal of the association between PTH and height development in XLH pediatric patients is best explained by conventional therapy. While initially lower PTH is associated with enhanced growth (2–6 years), higher values are associated with this in older individuals. However, this may be indicative of emerging secondary hyperparathyroidism in patients undergoing long-term therapy [47]. Elevated PTH levels are common in XLH patients and are due to mild hypocalcemia caused by FGF23-mediated impaired synthesis of 1,25 dihydroxy vitamin D as well as treatment with phosphate salts [1]. Thus, the positive association between linear growth and PTH levels in adolescence in the present analysis may indirectly reflect the (limited) growth promoting properties of phosphate treatment.

Increased risk for obesity in children with XLH is defined as a general problem in patients treated with phosphate salts and active vitamin D, even the positive association between body mass index (BMI) and treatment duration [13]. Actually, we found that body weight *z*-score decreased during early childhood which can be explained as the expected reaction on treatment with phosphorus supplements in age 1–5 years and active vitamin D [13]. However, there was a significant increase in body weight *z*-score with age during adolescence, reaching normal values at adult age, which suggests obesity in the face of poor linear growth. The only connection of lower phosphate and higher weight in later childhood and earlier age at diagnosis in adolescence suggests that the observed weight gain in patients with XLH may be due to further parameters (reduced physical activity, caused by rickets-associated pain and leg deformities) that were also not included in our analyses. In addition, a detailed genetic evaluation to determine potential associations between genotype and growth metrics in patients with XLH was unavailable. This recently became a limitation of our study.

It is also assumed that the head circumference as a biological metric is predominantly established in infancy, analogous to the previously mentioned body length. A lower parental height has been found to be associated with a larger head circumference in patients aged 6 to 12 years. However, this association indicates pathological changes with higher *z*-scores compared to a healthy population. The extent to which higher serum levels of phosphate and ALP in this age group and lower levels in adolescence are associated with larger head circumference remains unclear.

In summary, newborns with XLH have a 2.4-fold increased risk to be born SGA compared to healthy newborns, with smaller anthropometric characteristics at birth in case of an affected parent. They show body disproportion with reduced body length compared to body weight and head circumference, which further progresses during infancy, resulting in a significantly reduced body length and a larger head circumference compared to healthy children by the age of 2 years. This characteristic growth pattern may be used to identify children with XLH during routine medical check-ups. Supplementation therapy does not prevent progressive growth failure (which occurs mainly in infancy) and markedly reduced adult height in children with XLH.

## Supplementary Information

Below is the link to the electronic supplementary material.

Graphical abstract (PPTX 131 KB)

## Data Availability

The data supporting the findings of this study are available on request from the corresponding author. Data are not publicly available due to privacy or ethical restrictions.
