# Supplementary material for: Anthropometric characteristics at birth and growth outcome in patients with X-linked hypophosphatemia treated with oral phosphate and active vitamin D
Source: Pediatr Nephrol. 2026 Apr 10;41(9):3033–46. doi: 10.1007/s00467-026-07271-0 (PMC13424725; doi:10.1007/s00467-026-07271-0)
Supplement: Supplementary file 1 — Graphical abstract (PPTX 131 KB) [file 467_2026_7271_MOESM1_ESM.pptx]

## Slide 1
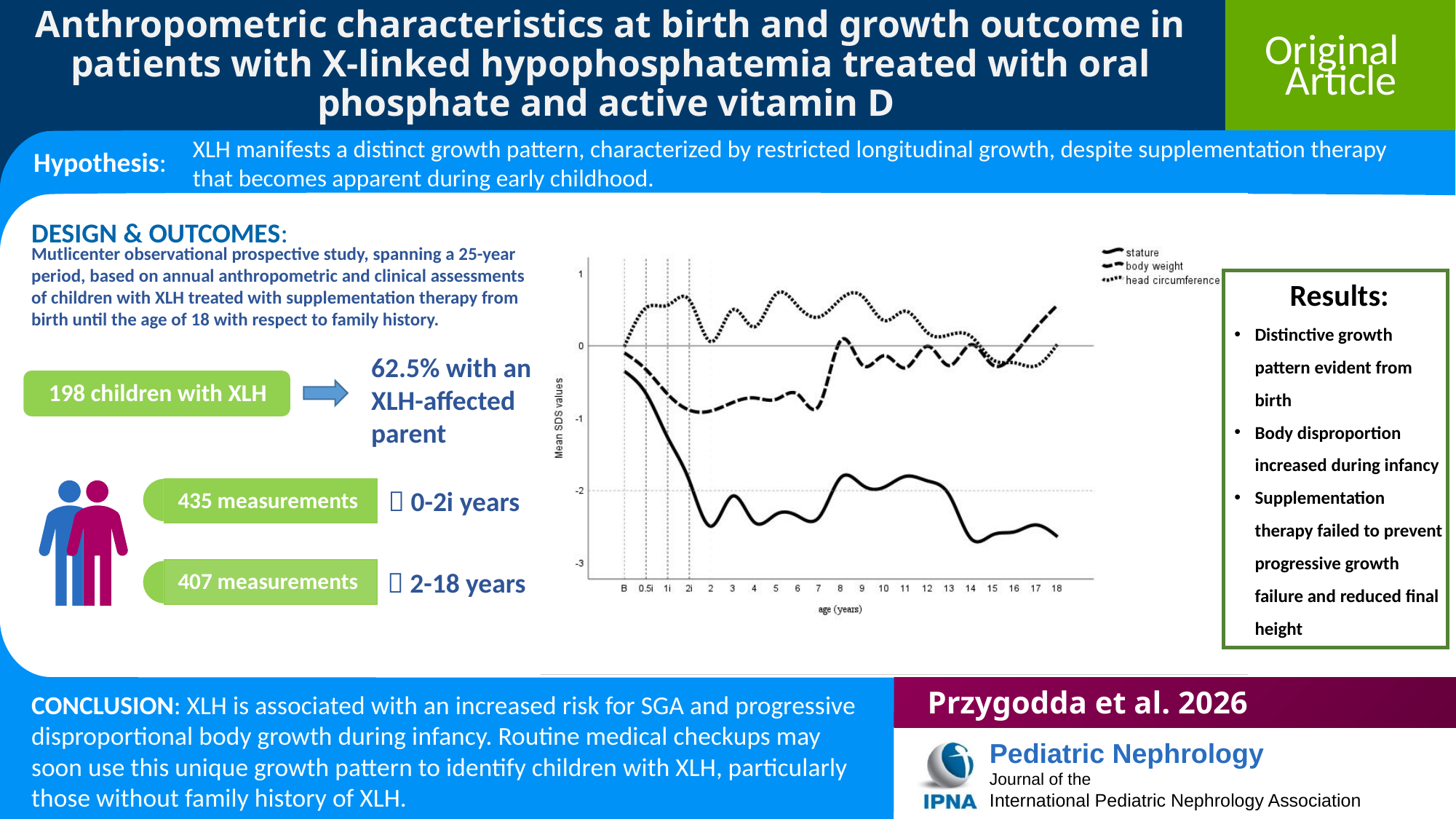

Anthropometric characteristics at birth and growth outcome in patients with X-linked hypophosphatemia treated with oral phosphate and active vitamin D
XLH manifests a distinct growth pattern, characterized by restricted longitudinal growth, despite supplementation therapy
that becomes apparent during early childhood.
Hypothesis:
DESIGN & OUTCOMES:
Mutlicenter observational prospective study, spanning a 25-year period, based on annual anthropometric and clinical assessments of children with XLH treated with supplementation therapy from birth until the age of 18 with respect to family history.
Results:
Distinctive growth pattern evident from birth
Body disproportion increased during infancy
Supplementation therapy failed to prevent progressive growth failure and reduced final height
62.5% with an
XLH-affected
parent
 0-2i years
 2-18 years
Przygodda et al. 2026
CONCLUSION: XLH is associated with an increased risk for SGA and progressive disproportional body growth during infancy. Routine medical checkups may soon use this unique growth pattern to identify children with XLH, particularly those without family history of XLH.
